# Software Design Challenges in Time Series Prediction Systems Using Parallel Implementation of Artificial Neural Networks

**DOI:** 10.1155/2016/6709352

**Published:** 2016-01-03

**Authors:** Narayanan Manikandan, Srinivasan Subha

**Affiliations:** School of Information Technology & Engineering, VIT University, Vellore, Tamil Nadu 632014, India

## Abstract

Software development life cycle has been characterized by destructive disconnects between activities like planning, analysis, design, and programming. Particularly software developed with prediction based results is always a big challenge for designers. Time series data forecasting like currency exchange, stock prices, and weather report are some of the areas where an extensive research is going on for the last three decades. In the initial days, the problems with financial analysis and prediction were solved by statistical models and methods. For the last two decades, a large number of Artificial Neural Networks based learning models have been proposed to solve the problems of financial data and get accurate results in prediction of the future trends and prices. This paper addressed some architectural design related issues for performance improvement through vectorising the strengths of multivariate econometric time series models and Artificial Neural Networks. It provides an adaptive approach for predicting exchange rates and it can be called hybrid methodology for predicting exchange rates. This framework is tested for finding the accuracy and performance of parallel algorithms used.

## 1. Introduction

The universal acceptance of agile methodology provides plenty of evidences in need for rapid adaptation in the current software development life cycle models. However, a number of recent leanings illustrate that a more all-inclusive approach is necessary rather than focusing on continuous integration of software.

Modern time series forecasting involves exchange rates prediction. Many factors that are correlated with each other in a way, namely, economic factors, political factors, and even psychological factors affect the foreign exchange rates by interacting in a complex fashion. Hence, the exchange rates are noisy, chaotic, and nonstationary. But research has shown that nonrandom and predictable behaviour can be emphasized in liquid market areas such as foreign exchange market.

There is a strong dependency between future exchange rates and that of the past. For more than two decades, Box-Jenkins ARIMA was used for time series data forecasting and widely used to benchmark other models. However, it has an assumption that the time series forecasting is linear and stationary by nature. So it results in a need to create a nonlinear model to be used in the prediction exchange rates.

Soft computing techniques are used for predicting currency exchange rates because of the function approximating nature. Parallel Artificial Neural Networks (PANN) in prediction and modelling are used. The multivariate analysis predicts future behaviour and other indicators such as technical, economic, and social indicators are combined along with the time series data in the forecasting process. ANN is more appropriate for the time series forecasting problem because of its nonparametric and adaptive properties. Research has shown that ANNs can map any nonlinear function without any prior assumption about the data. Earlier research in this area has proven that simple technical indicators are enough to obtain useful predictions and significant paper profit, without using any extensive knowledge or data related to the market.

**Figure 1:**
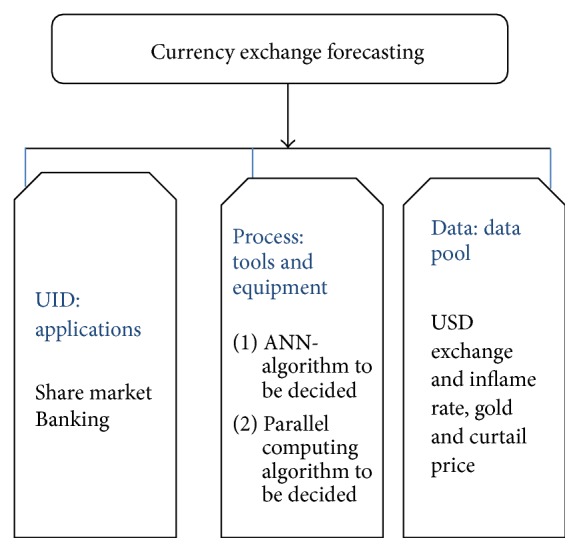
First-level design decisions.

This paper proposes a Heterogeneous Software design mythology in Figure 1 for forecasting currency exchange prices using Artificial Neural Networks. The same design will be validated by implementing of performance improvements through parallel computing. This model uses lagged time series data along with the technical and economic indicators as inputs and the predicted exchange rates of the desirable currency as output. This work results in forecasting the exchange rates between USD and five major currencies, namely, GBP, JPY, CAD, AUD, and EUR, using Back-Propagation Neural Networks. The Error Back-Propagation Network (EBPN) is trained with parameters like currency exchange rates, gold rates, crude oil rates, and US Inflation Rates for the period of January 2001 to March 2015.

Then the same design is modified without affecting its generality. With the help of Parallel Random Access Machine (PRAM), all processors act in lock-step; that is, the number of processors is not limited and all processors have local memory and one global memory accessible to all processors. Read and write operations are done on global memory and every processor involved in transaction knows its own index. Training and prediction can be done in CPU and GPU (graphics processing unit) based environment to improve the performance.

This work modified the existing scalar algorithms to address design reusability related issues and exploiting naturally parallelisable parts of existing algorithm and injecting brute force methods in each processor to use different initial conditions.

Further organisation of this paper is as follows: Section 2 describes related works and researches in software design, ANN, and parallel processing in the view of performance improvements.  Section 3 describes implementation issues, Section 4 shows results and discussion, and Section 5 describes conclusion and future work.

## 2. Related Works

Major focus is to explain lack of thinking between value and reducing waste. Any product piece or development pace that does not result in adding value is considered as waste. In software design adding and removing appropriate component result in efficiency parameters.

Much research and software development has been going on in forecasting the currency exchange prices in Forex market. ANN based implementation is familiar because of its nonlinear, predictive, and adaptive capabilities. When the amount of training and prediction data increased there is a definite need for parallel processing. In this section some related works done on ANN are presented, Forex market and parallel algorithms that are useful in decision making for effective design.

Useful prediction and significant profit can be made with simple technical indicators without the use of extensive market data or knowledge [1–3].

The author used daily time series data as input and used it to predict the Euro-USD exchange rate using genetic algorithm with Artificial Neural Networks up to three days ahead of the last data available. He used both macroeconomic variables and market data for inputs from which it was learnt that the exchange rate of Euro-USD was conditional. Some of the researchers have shown that a few technical indicators influence the exchange rates strongly. The said indicators are Nasdaq Index, Gold Spot Price, average returns of the government bonds, and crude oil price [4].

After much research using linear and nonlinear models, ANNs are said to perform better than ARIMA model in forecasting foreign exchange rates due to the nonlinear nature of the time series data. Some researchers have used the delayed time series data as input, while few other researchers have used Moving Average (MA) of the time series data as the input [2]. These works [2, 4]impressed us to decide major working components in this design.

The currency exchange rate data in the Forex market was said to be chaotic, random, and noisy in nature. In earlier days, the Random Walk Model and the Efficient Market Hypothesis were the two most widely used models based on fundamental analysis Certainly Forex data was noisy and random. But then, research through statistical tests showed with a significance of 95% that the Forex rates time series are not randomly distributed. To the neural networks since the Moving Average data tends to be a smoothed version of the delayed time series and with much less noise. Also, the MA technique is said to perform well only when the market follows a trend. However, it performs poorly when the index changes direction [5]. This work helps us in analysing feasibility of constructing data model for software.

With the help of above analysis, trade relation, and cost of imports and exports [2], to tackle the market evolution, the input data should be kept consistent. Otherwise, after training, the network is said to degrade in performance. One way to achieve consistency is to periodically replace the past data with recent data [5, 6]. Increasing the number of inputs is said to have not much effect on improving the performance [2].

As [7] specified in his work, the best activation function that can be used in the neural network design for prediction of time series data is a bipolar function [−1, 1] or a binary function [0, 1]. Reports [6–8] suggest that performance of the network does not improve when more than 2 hidden layers are used in the network. It has been reported that the presence of more than 2 hidden layers only makes the network more complex. It also makes the training process difficult and a danger of overfitting is present in such networks having more than 2 hidden layers. All the works in the neural network area have suggested the use of a maximum of 2 to 3 hidden layers as the optimum method to extract the best performance from the network.

To capture the regularities [9] proposed a moving window model that uses a two-layer back-propagation network with a fixed number of inputs modelling a window along the time series in fixed steps to capture the regularities in the underlying data [2]. For a large scale problem back-propagation learns very slowly and convergence is largely dependent on choosing suitable values of learning rate, momentum factor, and step size. Literature review revealed that the testing and validation set should be exactly one-fourth to one-eighth of the training set [5]. Kaastra and Boyd suggests a balanced split 70-15-15 for training, validation, and testing sets. Sigmoid function commonly used transfer function since the time series data is nonlinear in nature [6]. However some researchers have suggested the use of hypertangent function and tangent function too, as transfer functions [4, 8].

Usually the Normalised Mean Square Error is the most widely used metric [2, 3, 7] to measure the efficiency and the correctness of the trained neural network during the testing and validation process. But some researchers [2, 7] have used few other metrics too, in order to compare the performances and get the network to perform the best at any given situation. The error metrics that are used alongside NMSE to measure the correctness of the trained network are MAE (Mean Absolute Error), DS (Directional Symmetry), CU (Correct Uptrend), CD (Correct Downtrend), PMAD (Percentage Mean Absolute Deviation), RMSE (Root Mean Squared Error), MAPE (Mean Absolute Percentage Error), and MAE (Mean Absolute Error). Some have also reported using hit rate as a measure of correctness of a network [3]. Based on performance, it was reported that having small NMSE in validation and testing is more important than having small NMSE for training [3].

Using models based on Artificial Neural Networks, reports show that a correctness of up to 76% has been achieved in the earlier works with the variants of back-propagation algorithm as the learning method [1, 3].

In this work a fully pipelined parallel architecture exploits “mini-batch” training that combines different input cases to compute every set of weight updates to accelerate the power of ANN. Authors implemented this in FPGA; training mechanism is fully implemented in parallelised manner and obtains 100-time performance in running on a Virtex-6 LX760 FPGA [10].

Authors investigated in examining spatial optimization strategies like land allocation and planning will often require multiple data layers and complicated algorithms. It also deals with dynamic processes and the complicated relationships with massive amount of data. Authors developed a parallel geospatial model over the heterogeneous computer architecture of multiple CPU and GPUs. Experiments done with the data sets of California land details resulted in overall computing time for data collected in 50-year simulation which was dropped from 13,000 seconds on a single CPU into 32 seconds using 64 GPU/CPU nodes [11] which gives motivation for us to carry out this work.

In this paper ESR (Evolutionary Swarm Robotics) is an artificial approach for developing collective behaviour of homogenous autonomous robots. Its behaviour is generally controlled by evolving Artificial Neural Networks. However, ESR is unacceptable due to its very high computational cost. Through a detailed study, authors introduced a novel implementation to overcome the computational cost problem. A parallel algorithm for graphics processing unit (GPU) and OpenMP based solutions for multicore CPU. In this approach considerable performance improvement was achieved [12]. To apply parallelisms in currency exchange rate prediction related to Business Intelligence, the following works are considered.

The cloud based Business Intelligence (BI) has been demonstrated with a simulation on OPNET. It is a cloud model with layered OLAP applications with the possibility of applying parallel queries on relational databases. But this work also stated some challenges in taking BI into the cloud because of the restrictions of service providers. So much importance should be given for coordination of elements in architectural design and deploying it to enable the layers of OLAP for better decision making, while designing a BI more significance has to be given for the resource management to avoid bottlenecks. Service providers should plan effectively on available details and implement the same based on infrastructure, platform, and application components to achieve a massively parallel processing system with the support of enhancement framework using all available technologies efficiently [13].

Victor Chang proposed Business Intelligence in cloud based services is very much useful work with respect to predictions in Business Intelligence. Proposed Business Intelligence as a service (BIaas) has Heston model for the investors to take decision before investing and author has used RMSE (Root Mean Squared Error), MA (Moving Average), and EWMA (Exponentially Weighed Moving Average) as calibration. Heterogeneous Software design methodology was used in this implementation [14].

In this work Ramachandran and Chang proposed financial software as a service in cloud environment. The architecture itself sounds good because of its heterogeneity and integrity among the components. Major part of implementations takes Monte Carlo methods, Black Scholes Models, and Variance Gamma process. Here Variance Gamma method is used for outlier removal. Highlights of this paper are MATLAB based implementation and focused towards achieving accuracy and performance in cloud environment [15].

## 3. System Implementation

### 3.1. System Description

First some important design decisions should be taken with the following assumptions.

The system is a forecasting model built using neural networks, where the input layer takes the input variables. Both technical and economic variables are taken as input. The hidden layers process the input variables and add value to the system. The hidden layers contain 20 neurons. The output layer yields univariate output. Then a parallel approach for training the algorithm is implemented in the system with the configurations stated in Table 3. This process analyses the impact on change management in software design.

The network is constructed by interconnection of artificial neurons. Different components used in building the parallel forecasting model are as follows.

*(i) NAR Network to Predict Input Variables*. This section determines prediction details on ask prices of USDJPY, USDAUD, USDCAD, USDCHF, and GBPUSD currencies, respectively, as well as prediction of gold price, crude oil price, CPI, and inflation rate.

The NAR (Nonlinear Autoregressive) network takes the input variable *y*(*t*), where the network is trained with the past values of time series to predict its future values. In this example, this approach predicts the variables like ask prices of the five currencies, gold and crude oil price, CPI, and the different inflation rate.

**Figure 2:**
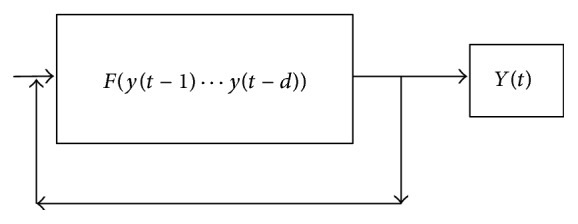
Design for input data generation.

Considering Figure 2, once the network is trained with past values, with the help of parallel algorithms values are predicted from point of prediction and up to the threshold level of expected accuracy.

**Figure 3:**
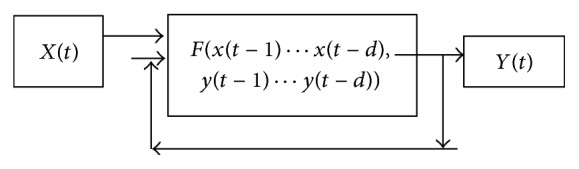
Design of output prediction using NARX.

*(ii) NARX to Predict the Output Variable*.  Figure 3 shows the NARX network that is used for the prediction of the bid price of the desired currency to be forecasted, with the NAR network predictions applied as input to the trained network.

The NARX network takes the input variables as exogenous inputs that all the 10 input variables are taken for input in this network, and the network is trained by assigning the time series output variable that this technique needs to predict as the target variable. Once the network is trained using exogenous input and required output, then, all the trained NAR network predictions of input variables can be supplied to this network as inputs to predict the target output with the presence of delay. This process gives Number of values from the point of prediction to predict the next required number of steps.

Usually the performance degrades with every step of prediction since predicted values are used as feedback to targets instead of original values and it can be solved by using parallel algorithms in appropriate places. Therefore a threshold is set to predict until a particular number of steps to reach good amount of accuracy. NARX network is trained with 25 neurons in hidden layer with the delay of 20.

Number of neurons in this technique is considerably high because of the high processing requirements for data such as a financial time series. Reason for delay of 20 is high correlation because of target values existing in data set (values of one month before the current value used in both input and target variables). This can be reduced by altering design with utilization of parallel resources.

NARX network uses “trainlm” (a predefined algorithm function name, for Levenberg-Marquardt back-propagation algorithm) to train the network. Mean Squared Error (MSE) is used as the performance measure, calculating the mean squared difference between the expected target output and the actual output.

*(iii) Time Series Data*. MATLAB based implementations are used to promote the reusability for training and prediction process. Excel file is imported using data import Interface and appropriate data for training and Predictions are selected.

Once the required data is selected as in Table 2, it is then converted into either of the following formats, to facilitate the network construction process:

- Matrix.
- Cell Array.
- Column Vectors.

In general, Matrix or Cell Array representation is used to store data in the workspace.

The extracted data that is used for training, validating OR predicting, are all stored in the workspace in MATLAB file format with the extension  .mat.

This MATLAB file can be accessed when a data is required; this is a simple way of storing data, because all of the data involved is numerical in nature and contains time steps of time series data. There will be less usage if repository based architecture was chosen.

**Figure 4:**
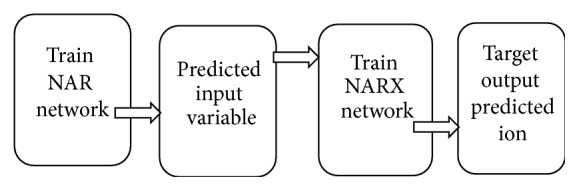
Dependency network.

*(iv) Architecture Design for Handling Dependencies*. The NARX network depends on the other NAR network predictions for its input as it uses those predicted inputs to support the input layer and to predict the target output.  Figure 4 represents ancient pipes and filter architecture style which is specialized in designing applications with dependencies.

*(v) Choosing Appropriate Algorithm for Low Level Design: Parallel Back-Propagation Learning Approach*. This section elaborates the low level design decisions. First Levenberg-Marquardt (LM) algorithm is the most widely used algorithm for optimization. It works well in simple gradient descent and conjugate gradient methods in varieties of problems. LM algorithm gives solutions in the form of* Nonlinear Least Squares Minimization*. The LM algorithm blends speed advantage of Gauss-Newton (GN) algorithm and stability of the steepest descent method. But it is more robust than the GN algorithm, because in many cases it can handle well even if the error surface is much more complex than the quadratic situation.

LM was designed to achieve second-order training speed without calculating Hessian matrix values.

Performance function has sum of squares; Hessian matrix is approximated as

(1) $$\begin{array}{c} H_{m}=J^{T}J. \end{array}$$

Gradient can be computed as

(2) $$\begin{array}{c} G=J^{T}e, \end{array}$$

where  *J* is Jacobian matrix containing first derivatives of the network errors based on the weight and biase. *e* is vector of network errors.

The Jacobian matrix can be computed with less complexity than Hessian matrix through a standard back-propagation. The LM algorithm uses this approximation to the Hessian matrix in the following method called Newton-like update:

(3) $$\begin{array}{c} X_{k+1}=x_{k}{\left[\;j^{T}J+\mu I\;\right]}^{-1}J^{T}e, \end{array}$$

where scalar *μ* is zero, in Newton's method for using approximation Hessian matrix.

If *μ* is large in gradient descent with less steps, then value will be decreased after every successful step.

**Algorithm 1 (back-propagation training).**

 

*Initial Assumptions*. Consider the following:

 
*e*_th_: error threshold.
 
*T*_it_: target iteration.
 
*e*: error on output.
 
*o*: actual output.
 
*t*: target output.
 Hid: hidden layer.
 
*P*: current pattern.

(1) Divide the training data set *D* into equal parts *D*_1_, *D*_2_,…, *D*_*N*_, where *N* is the number of threads in process;
(2) initialize the weights, Desired error threshold *e*_th_ and Total iterations *T*_it_
(3) Channel weight = 0; iteration = 0;
(4) *i* = size of available patterns;
(5) while *i* not equal to zero;
(6) Calculate Error in output neuron *e*_*i* = 0_*i* − *t*_*i*;
(7) Calculate Hid_*e*_*i* = *e*_*i∗*Weight_hid*∗*Derivative_*y*  (*P*). We_hid. Derivative_*y* (*P*); // derivative function for *y*
(8) Calculate the channel weights *W*_*ij*_ for output neurons and hidden layer
(9) Accumulate the newly calculated weight in initial weight for ever iterations.
(10) End while
(11) Update weights in the network based on learning rate
(12) Calculate *e*_out_-MSE on training data *D*,
(13) if *e*_out_ > *e*_max_ and current iteration > maximum number of iterations increment the iterations and continue.

With the help of Algorithm 1, LM back-propagation algorithms can be parallelised, and batch training approach is used. Since batch training is relatively easy to adapt for multithreaded and multicore CPUs, Steps (4) to (10) can run in threads to perform back-propagation in parallel on different patterns. For each pattern *p*_*i*_ weights and errors can be separately calculated and stored.

**Figure 5:**
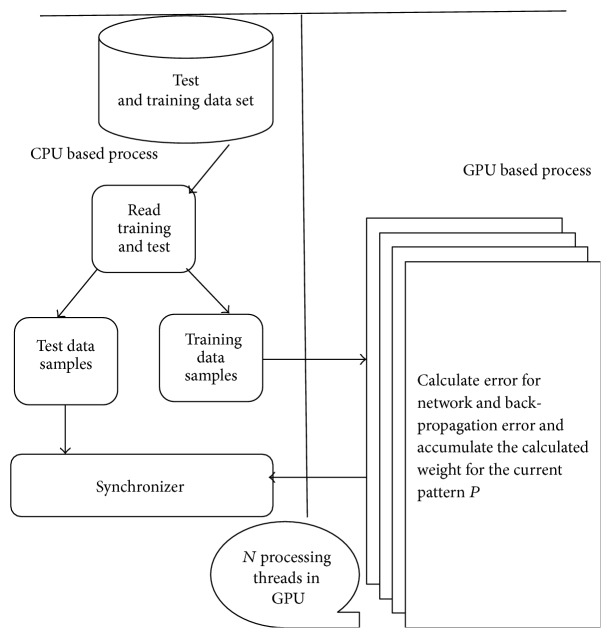
Hybrid architectural designs for training.

Figure 5 represents the high level architecture of training process. Here major responsibility is assigned to synchronizer module that performs the following tasks:

- Wait till all threads completes the training process.
- Calculate overall weight for the network.
- Calculate network error for test data.
- Iterate the above process until all patterns are trained sufficiently.
- To stop the training process this approach has identified following conditions.

When the maximum number of iterations is reached or maximum amount of time is exceeded, performance is minimized to goal due to performance gradient below min_grad.

In the first step, a time series model based on Artificial Neural Networks generates the estimates of the currency exchange rates and other technical parameters that are used to forecast the exchange price of currency of our choice. In the second step, Error Correction Back-Propagation Neural Network is used to correct the errors of the estimates. The proposed two-step model produces better accuracy in results than the single step models.

## 4. Results and Discussion

First Heterogeneous Software design merits are given in Table 1.

Ramachandran and Chang and Muntean et al. [15, 16] also used heterogeneous designs for prediction based systems and the results sound good.

Implementation is done with the following steps:

1. Collected data (as per Section 4.1) is stored in Excel file.
2. Use NTSTOOL of MATLAB neural network time series tool. With NTSTOOL NAR, NARX, and Levenberg-Marquardt algorithms are implemented.
3. Then implemented Algorithm 1 (parallelised LM algorithm) in MATLAB with parallel processing capability and execution time of each training is noted and compared.

The following sections describe data collections, performance metrics used, and results of implementations.

### 4.1. Data Collection

The data used in this analysis are the daily foreign exchange rates of five currencies against US Dollar along with prices of crude oil, gold, US Inflation Rate, and CPI from the period of June 1993 to March 2015 made available by Oanda.com. This approach took into consideration the exchange rates of Australian Dollar (AUD), British Pound Sterling (GBP), Canadian Dollar (CAD), Swiss Franc (CHF), and Japanese Yen (JPY).

### 4.2. Construction Of Performance Metrics

Performance of the above forecasting model is evaluated with the help of three statistical metrics.

Mean Squared Error (MSE) is as follows:

(4) $$\begin{array}{c} MSE=\frac{1}{n}\overset{n}{\underset{i=1}{\sum }}{\left(\;{\hat{Y}}_{i}-Y_{i}\;\right)}^{2}. \end{array}$$

Mean Absolute Error (MAE) is as follows:

(5) $$\begin{array}{c} MAE=\frac{1}{n}\overset{n}{\underset{i=1}{\sum }}\left|\;f_{i}-Y_{i}\;\right|=\frac{1}{n}\overset{n}{\underset{i=1}{\sum }}\left|\;e_{i}\;\right|. \end{array}$$

Sum Squared Error (SSE), also otherwise called Residual Sum of Squares (RSS), is as follows:

(6) $$\begin{array}{c} RSS=\frac{1}{n}\overset{n}{\underset{i=1}{\sum }}{\left(\;Y_{i}-f\left(\;x_{i}\;\right)\;\right)}^{2}. \end{array}$$

Here, MSE and MAE measure the deviation between the actual value and the predicted value. SSE is a measure of discrepancy between the data and the forecasting model.

Higher accuracy of prediction is indicated by the presence of smaller MSE and MAE values.

*Performance Measures for Parallel Algorithms*. Consider the following:

(7) $$\begin{array}{c} Efficiency=\frac{\in_{single}}{N\cdot \in_{parallel}}, \end{array}$$

where ∈ is  execution time.

*Assumptions*. Single threaded and parallel trainings are initialized with same network weights for training. The experiment is repeated 15 times by changing network configurations. This algorithm is tested with the following Intel based computer.

### 4.3. Simulation Results

To implement this, at the point of prediction, the predicted value is given as feedback instead of using the original input values; closed loop back-propagation design is chosen. All NAR networks are trained with a maximum of 35 neurons in hidden layer with delay of 20. The maximum performances achieved around 19 to 25 epochs. Results were shown in Table 4.

The “trainlm” (a predefined algorithm function name, for Levenberg-Marquardt back-propagation algorithm) is used to train the network. And Mean Squared Error (MSE) is used as the performance measure, calculating the mean squared difference between the expected output and the actual output.

A NARX neural network model was trained with 7 technical indicators and 2 economic indicators, a hidden layer, and an output neuron unit to predict the exchange rate. The network uses Levenberg-Marquardt training algorithm which adaptively changes weights during each back-propagation and the training is stopped when the best performance for the given inputs and output is obtained for both training and validation. The number of hidden neuron units was modified between 15 and 20 and the training was terminated at epochs between 60 and 100.

**Figure 6:**
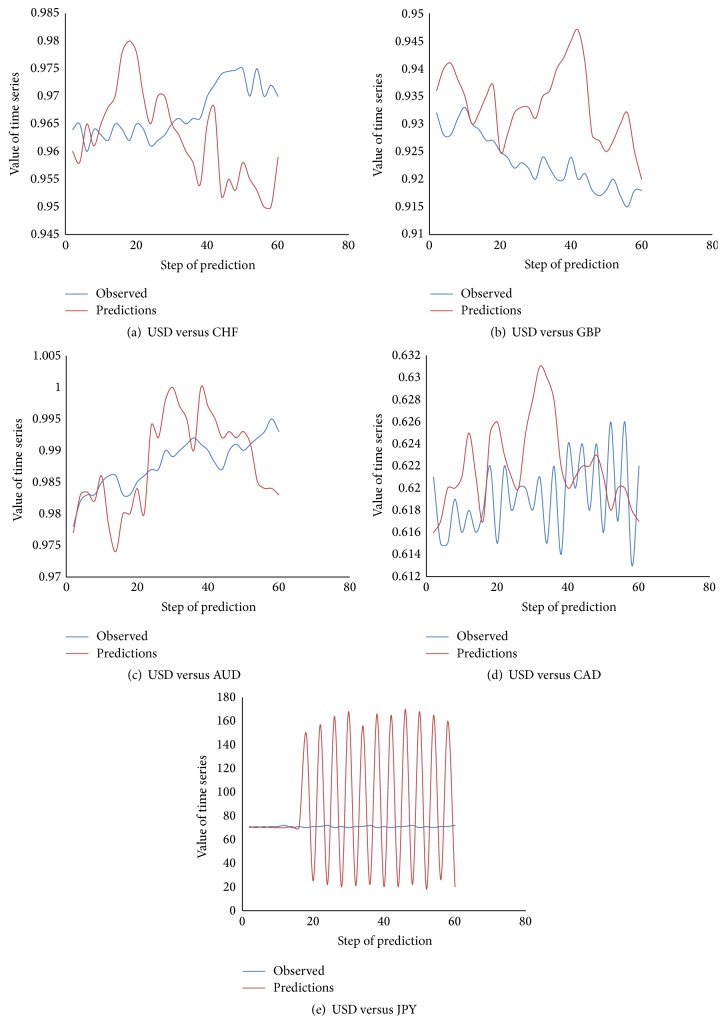
Implementation results of predictions.

Based on the performance metrics measurements performed on the predicted data, this approach found out that the trained networks gave the best performance predictions with high rates of accuracy for GBP, CHF, AUD, and CAD for 60 days from the point of prediction. But for the JPY currency exchange rate, the prediction accuracy lasted for only 15 days from the point of prediction which is shown in Figure 6(e). This model is created for short term trend forecasting, hence 60-day period of prediction. The model can be extended to 120–150 days with minimum loss of accuracy. The Levenberg-Marquardt algorithm of back-propagation works well for this application when compared to the performance of the previous works in foreign exchange rates prediction.

The performance of such high accuracy is obtained due to the improved technique used for learning, in the Levenberg-Marquardt algorithm, combining the advantages of gradient descent and Gaussian-Newton methods. The MSE, MAE achieved by the trained NARX network is visibly higher than those obtained using other methods in the researches done in the past [2].

The diagrams comparing the actual and the predicted exchange rate of the five currencies are shown in Figures 6(a)–6(e). The plots show that the forecasting follows the actual rates more closely in the case of AUD, GBP, and CAD. For CHF and JPY the prediction is relatively closer to the actual rates.

From the above plots, performance for USD/JPY degrades after 15 days. The other 4 currencies' prediction shows significant accuracy around 90–95% for the first 120 days from the prediction start date and more than 97% accuracy for the first 60 days of the prediction which is significantly higher when compared the other models used in the previous researches [1, 3]. This means that, for improved accuracy, the network has to be retrained every 120 days.

### 4.4. Parallel Implementation of Same Algorithm

The proposed approach used MATLAB based parallel libraries to make use of library functions. It has implemented changes stated in Figure 5, a hybrid software architecture that addresses the following problems in the sequential execution:

- Increasing the execution time of training algorithms. It gives good impact on the overall execution.
- Utilizing maximum processing power of available resources.
- Reducing the cost of implementation through reducing execution time.

**Figure 7:**
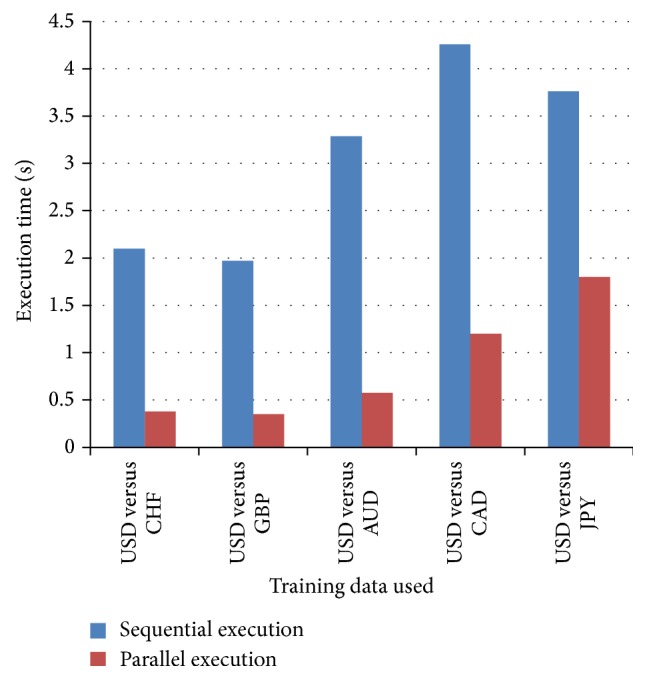
Performance improvement through parallel execution.

Same methodology is executed using parallel training approach. Based on (7), the improvements in execution time are obtained as shown in Figure 7.

## 5. Conclusion and Future Work

This analysis gave rise to the following conclusions. Heterogeneity based software design is more suitable for soft computing based applications and introducing parallel algorithms at any possible stages will increase the performance. The prediction results are significantly promising for the four currencies GBP, AUD, CAD, and CHF. The prediction performance for Japanese Yen is very poor. Instead of using MSE alone, the proposed approach used two other metrics along with that to measure the performance of the network. But other additional metrics can be used to significantly measure the performance which can be used for comparisons.

The Levenberg-Marquardt back-propagation algorithm that is used in this study to build and train the network has proved to be worthy in combining technical and economic indicators to perform the prediction.

The above observations have confirmed the better performance of Artificial Neural Networks in the forecasting of currency exchange rates.

Further research emphasis will be on using just the technical indicators in the NARX network and obtaining a performance better than the models that were previously used for the purpose of forecasting the currency exchange rates, using the LM algorithm that was used for this study.

## Figures and Tables

**Table 1:** Quality attributes of software design.

Factors of heterogeneous design	Medium	High
Simplicity		High because well-known components of different technology can be used together

Portability	This design will not fit for implementation in all kinds of platforms	

Modifiability		Actual design and modified design produced the same results

Reliability	Except Japan currency versus USD other predictions are reliable	

**Table 2:** Data collected for analysis.

Process number	Number of daily data	Number of training data	Number of test data
1	48100	33670	14430
2	58200	39500	18700

**Table 3:** CPU and GPU configurations used.

Processor number	i5-6440HQ
Intel Smart Cache	6 MB
DMI3	8 GT/s
Instruction set	64-bit
Number of cores	4
Number of threads	4
Processor frequency	2.6 GHz
Processor graphics	Intel HD Graphics 530
Graphics frequency	350 MHz
Graphics maximum dynamic frequency	950 MHz

**Table 4:** Measurement of prediction performance over 60-day prediction.

Currency	Performance metrics		
	MSE	MAE	SSE
Australian Dollar	9.74*E* − 05	7.64*E* − 03	3.45*E* − 03
British Pound	2.35*E* − 05	3.96*E* − 03	8.94*E* − 04
Canadian Dollar	3.20*E* − 05	4.29*E* − 03	1.23*E* − 03
Swiss Franc	1.10*E* − 04	8.62*E* − 03	4.31*E* − 03
Japanese Yen	2.21*E* + 03	2.83*E* + 01	9.65*E* + 04
